# Genomic and phenotypic analysis of invasive *Streptococcus suis* isolated in Spain reveals genetic diversification and associated virulence traits

**DOI:** 10.1186/s13567-024-01267-0

**Published:** 2024-01-24

**Authors:** Cristina Uruén, Ana Fernandez, José Luis Arnal, Mateo del Pozo, Maria Casas Amoribieta, Ignacio de Blas, Paula Jurado, Jorge Hugo Calvo, Marcelo Gottschalk, Luis Daniel González-Vázquez, Miguel Arenas, Clara M. Marín, Jesús Arenas

**Affiliations:** 1 Unit of Microbiology and Immunology, Faculty of Veterinary, University of Zaragoza, Zaragoza, Spain; 2 Institute Agrofood of Aragón-IA2, University of Zaragoza-Center of Research and Technology of Aragón (CITA), Zaragoza, Spain; 3 Exopol. Veterinary Diagnostic and Autogenous Vaccine Laboratory, San Mateo de Gállego, Zaragoza, Spain; 4 Labopat, LABOPAT NUZOA SL, Segovia, Spain; 5 Ovislab, Barcelona, Spain; 6 Unit of Infectious Diseases, Faculty of Veterinary, University of Zaragoza, Zaragoza, Spain; 7 Department of Animal Science, Center of Research and Technology of Aragón CITA, Zaragoza, Spain; 8 ARAID, Saragossa, Spain; 9 Research Group on Infectious Diseases in Production Animals and Swine and Poultry Infectious Diseases Research Centre, Faculty of Veterinary Medicine, University of Montreal, Saint-Hyacinthe, QC Canada; 10 Department of Biochemistry, Genetics and Immunology, University of Vigo, Vigo, Spain; 11 CINBIO, Vigo, Spain

**Keywords:** *Streptococcus suis*, epidemiology, virulence factors, genetic recombination

## Abstract

*Streptococcus suis* is a zoonotic pathogen that causes a major health problem in the pig production industry worldwide. Spain is one of the largest pig producers in the world. This work aimed to investigate the genetic and phenotypic features of invasive *S. suis* isolates recovered in Spain. A panel of 156 clinical isolates recovered from 13 Autonomous Communities, representing the major pig producers, were analysed. MLST and serotyping analysis revealed that most isolates (61.6%) were assigned to ST1 (26.3%), ST123 (18.6%), ST29 (9.6%), and ST3 (7.1%). Interestingly, 34 new STs were identified, indicating the emergence of novel genetic lineages. Serotypes 9 (27.6%) and 1 (21.8%) prevailed, followed by serotypes 7 (12.8%) and 2 (12.2%). Analysis of 13 virulence-associated genes showed significant associations between ST, serotype, virulence patterns, and clinical features, evidencing particular virulence traits associated with genetic clusters. The pangenome was generated, and the core genome was distributed in 7 Bayesian groups where each group included a variable set of over- and under-represented genes of different categories. The study provides comprehensive data and knowledge to improve the design of new vaccines, antimicrobial treatments, and bacterial typing approaches.

**Supplementary Information:**

The online version contains supplementary material available at 10.1186/s13567-024-01267-0.

## Introduction

*Streptococcus suis* is a Gram-positive bacterium that has emerged as a leading cause of large economic losses in the porcine industry worldwide. As a commensal, it colonizes the upper respiratory tract of neonatal pigs up to 100% in most farms. Virulent strains can access the bloodstream and cause a systemic infection, including septicaemia with sudden death, meningitis, arthritis, and/or endocarditis, among others [1]. The disease affects pigs at different ages but suckling and (mostly) weaning pigs (up to 12 weeks old) are the most susceptible. In a recent epidemiological study performed in a few EU countries, including Spain, about 60–80% of the farm units are clinically affected by *S. suis* [2]. In addition, *S. suis* is a zoonotic agent, traveling from animals to humans by different routes, with a high prevalence in some Asian countries.

*S. suis* infections are mostly treated with antibiotics. Actually, the high incidence of the disease forces an extensive use of antibiotics, sometimes administrated as an early metaphylactic therapy. But this practice has contributed to the global emergence of multidrug-resistant strains [3]. Also, vaccination strategies were proposed to fight streptococcal infections [4]. The most broadly used strategy in the field involves inactivated whole-cell autogenous vaccines, i.e., bacterins. However, their efficacy is controversial [5]. One of the main drawbacks is the large genetic variability of the bacterium that prevents cross-protection against new genetically divergent clones. In addition, the loss of epitopes during the inactivation process, the difficulties in isolating the causal clone, or the lack of effective vaccination programs contribute to reduced vaccine efficacy [4].

Capsular polysaccharide is a major virulence factor and has been proposed as a relevant antigen for vaccinology and molecular typing. Based on capsule nature, up to 35 capsule types (serotypes) were associated with *S. suis* [1], but few were later reclassified. Serotypes 2, 9, 1/2, and 3 are the most prevalent worldwide [1], but also large differences can be observed in particular geographic regions. In this regard, Multi-locus Sequence Typing (MLST), which provides an alternative to serotyping [6], can distinguish among many genotypes and allows global and long-term epidemiology for many bacteria. A MLST scheme is publicly available for *S. suis* and more than 3800 Sequence types (STs) were identified so far.

Spain is one of Europe’s highest pig production countries, with a production of about 30–35 million heads per year. Despite its economic relevance and the high incidence of *S. suis*-disease in Spain [2], the number of genomic studies is limited [7–10]. Understanding the genetic variability and dispersion of current invasive isolates is the basis to design and develop effective diagnostic and vaccinology approaches. Hence, here we aimed to analyse the genetic architecture of pig-invasive* S. suis* circulating in Spain.

## Materials and methods

### Bacterial isolates and growth conditions

A panel of 156 *S. suis* invasive isolates recovered from pigs with clinical signs of streptococcal swine disease were studied here and their origin and characteristics are listed in Additional file 1. The isolates were selected from a total of 2453 *S. suis* isolates. The selective criteria were: (i) only one isolate from the same farm, to avoid over-representation of endemic clones. Politically, and territorially, Spain is organized in 17 Autonomous Communities of variable size and with certain managerial autonomy. Each Autonomous Community is organized in a variable number of provinces. (ii) isolates should represent the most productive Autonomous Communities. (iii) only isolates from internal organs or blood in which only one clone (serotype) was detected (to rule out the selection of opportunistic clones); (iv) isolates recovered from the period 2014–2021 to have an indication of the current circulating *S. suis*. A total of 843 isolates matched the selective criteria, from which 156 were selected following a random selective process. Additional file 2 summarizes the characteristics of the isolates related to the host. Bacterial growth conditions were as in previous studies [11, 12]. Shortly, bacteria were grown in BD™ Columbia Agar with 5% Sheep Blood (Columbia, Heidelberg, Germany) or Todd-Hewitt Broth or Agar (THB or THA, respectively, Oxoid Ltd., Hampshire, England) at 37 °C in a candle jar for 24 h. For bacterial liquid cultures, bacteria were propagated in THB at a starting Optical Density of 600 nm (OD_600_) of 0.2 and incubated overnight.

### PCR amplification and sequence type (ST) assignation

PCRs were performed using 1 µl of genomic DNA, 0.25 μM of primer combinations (Additional file 3), 0.5 U of Taq DNA polymerase, 400 μM dNTPs, and PCR buffer. All components were obtained from Biotools. PCR conditions were an initial step of 10 min at 95 °C, followed by 30 cycles of 1 min at 95 °C, 0.5 min at the annealing temperature described in Additional file 3, 2.5 min at 72 °C, and a final step of 10 min at 72 °C in a thermocycler (Biometra TRIO, Madrid, Spain). The PCR products were visualized on 1% agarose gels with Green^®^Nucleic Acid Stain (Sigma-Aldrich, Darmstadt, Germany) in a gel reader.

The *S. suis gdh* and *recN* genes were amplified following the previously described protocols [13–15], and used for the identification of *S. suis*. For serotyping, several genes coding for enzymes of the capsule biosynthesis pathway were amplified following the scheme previously described [16, 17]. For MLST, seven housekeeping genes, including *aroA* (coding for 5-enolpyruvylshikimate3-phosphate synthase), *cpn60* (coding for 60-KD chaperone), *dpr* (coding for a putative peroxide resistance protein), *gki* (coding for glucose kinase), *mutS* (coding for a DNA mismatch repair enzyme), *recA* (coding for homologous recombination factor) and *thrA* (coding for aspartokinase/homoserine dehydrogenase) were amplified following an established PCR protocol [18], but *mutS* gene was amplified using the protocol described by Rehm et al. [19]. For the identification of *epf*, *mrp* and *sly* genes, PCR conditions were used as described by [20]. In addition, 10 additional virulence-associated genes (VGs) were selected, which encompass surface exposed or secreted structures with a proposed involvement in virulence, including *hylA* (coding for hyaluronidase A) [21], *dppIV* (coding for dipeptidyl-aminopeptidase IV) [22], *zmp* (coding for specific Zinc metalloproteinase) [23], *sbp2 *(coding for putative pili subunit protein) [24], *sspA* (coding for subtilisin-like serine proteinase) [25, 26], *apuA* (coding for surface anchored amylopullulanase) [12, 27], SSU1773 (coding for surface anchored serine protease) [12, 28], *htpsC* (coding for histidine triad protein C) [29, 30], *ofs* (coding for serum opacity factor) [31], and *srtF* (coding for sortase F) [32]. In addition, several of these genes were used as epidemiological markers in different studies [12, 33, 34]. When required, the resulting amplicons were purified using the FavorPrep GEL/PCR Purification Kit (Favorgen, Ibian, Zaragoza, Spain) following the manufacturer’s instructions, quantified in Nanodrop, and sequenced at STAB Vida Lda (Caparica, Portugal).

PCR products were sequenced, and allelic profiles generated by sequencing of seven housekeeping genes were submitted to the *S. suis* MLST database website. Clonal complexes (CCs), which englobe STs that share similar sequence with at least 6 loci, were following previous literature assignments. To classify the STs into closely related phylogenetic groups, eBURST analyses were performed with the tool developed in PubMLST database, establishing groups when the profiles of 2 or more isolates matched at least 5 loci with any other member of the group.

### Whole genome sequencing and bioinformatics analysis

A total of 19 isolates of different STs were used for whole genome sequencing, including three of ST1 (Ss_21, Ss_22, Ss_72), two of ST123 (Ss_84, Ss_106), and one of ST3 (Ss_45), ST24 (Ss_24), ST1637 (Ss_124), ST1642 (Ss_134), ST16 (Ss_109), ST29 (Ss_20), ST1628 (Ss_53), ST949 (Ss_24), ST17 (Ss_46), ST1625 (Ss_48), ST1626 (Ss_51), ST1627 (Ss_52), ST1654 (Ss_107) and ST1637 (Ss_115). DNA-Seq libraries were prepared at the StabVida Lda (Caparica, Portugal), and sequencing was carried out with a HiSeq 2500 generating paired-end reads of 151 base pairs (bp) with an expected yield of 1 Gbp/isolate for whole genome DNA Sequencing using Illumina technology. Quality control of the raw and trimmed paired-end reads was performed with FastQC v.0.11.7. Trimming was performed with Trimmomatic v.0.38 [35] to remove low-quality reads and adaptors. Besides, microbial DNA was also sequenced using nanopore technology using a MinION sequencer (Oxford Nanopore Technologies, ONT). One μg of DNA from each sample was used for sequencing, following the ligation sequencing kit (SQK-LSK109) protocol. Nine and ten samples were multiplexed in two different runs with the 1D Native Barcoding genomic DNA kit (EXP-NBD104 and EXP-NBD114). The barcoded samples (700 ng of DNA in total) were pooled, and adapter ligation was performed for sequencing using two R9.4.1 flow cells. Base calling was prepared with the Guppy 4.2.2 software provided by ONT. Possible contamination by human DNA (*Homo sapiens* (b38):hg38) was removed with the bowtie2 tool. Finally, sequences were assembled with the SPAdes tool, matching the trimmed short-read Illumina sequences with those obtained by nanopore sequencing. Assembly quality was assessed using Quast 5.0.2 [36]. Genomes were annotated using PROKKA, and pan genomes were analysed with ROARY. FragGeneScan was also used to predict prokaryotic genes or open reading frames [37]. After this, functional annotation was performed using precomputed eggNOG-based orthology assignments in Eggnog-mapper [38]. Differential Abundance Analysis of Functions (EggNOG) implemented in OmicsBox was applied to detect which functional annotations are enriched between different conditions, in our study, the different Bayesian groups (BAPs). The statistical test of this tool is based on an over-dispersed Poisson generalized linear model. Thresholds were set in *p* < 0.05 for FDR and 2 and −2 for Fold Change. Finally, the core genes (previously obtained with Roary) were clustered into 7 groups using the BAPs [39] method implemented in RhierBAPS [40]).

DNA sequences were aligned with MAFFT [41] and a phylogenetic tree was calculated with MEGAX. When required, the non-synonymous/synonymous substitution rate ratio (dN/dS) was estimated with the single-likelihood ancestor counting (SLAC) method [42] implemented in HyPhy [43]. In addition, the RDP4 program [44] was used to detect recombination events, and their breakpoints, in the studied sequences. Mobile genetic elements (MGEs) were found with MGEfinder service offered by the Centre for Genomic Epidemiology [45], using all the obtained nodes.

### Statistical analysis

In a first approximation, the association between the most prevalent genotypes, isolation year, anatomical isolation site, age and symptoms of the animals, geographical location, serotypes, and presence of VGs, was assessed with the Pearson’s chi-square test, except in the analyses where more than 20% of the cells showed an expected frequency lower than 5 and in this case the likelihood ratio test (LRT) was used. For both tests, significant association between groups was considered when the *p*-value was lower than 0.05. To detect which categories were significantly related, we analysed the adjusted standardised residues (ASR). When the ASR score was higher than 1.96 the relationship was considered as positively significant (ASR is higher than the expectation), while if ASR was lower than −1.96 the association was considered as negatively significant (ASR is higher than the expectation). By contrast, if the ASR score was between −1.96 and 1.96 the association between groups was considered as not statistically significant. Next, to confirm the statistically significant associations detected with Pearson’s test and the LRT, 2 × 2 contingency tables were obtained using Fisher’s exact test, and scores with a *p*-value lower than 0.05 were considered as statistically significant. These statistical analyses were performed with SPSS v.26 software (IBM Corporation, Armonk, NY, USA).

## Results

### Isolate collection

We studied a panel of 156 invasive isolates of *S. suis* recovered from pigs with clinical signs of *S. suis* disease. The origin and characteristics of each isolate are listed in Additional file 1. The isolates were selected from a total of 2453 *S. suis* isolates. The selective criteria are indicated in Materials and methods. Additional file 2 summarizes the characteristics of the isolates related to the host. The most frequent anatomical site of isolation was the central nervous system (CNS) (53.8%) and joints (28.2%) (Additional file 2A). Most of the pigs exhibited a combination of clinical signs that mainly affected the nervous system (67.3%), and arthritis (49.4%), but also septicaemia, pneumonia, pericarditis, and sudden death (Additional file 2B). 9% of the isolates were obtained from suckling piglets (0–3 weeks old), 63.5% from nursery pigs (3–10 weeks old) and 1.9% from fattening pigs (more than 10 weeks old) (Additional file 2C). Considering the isolation year, isolates were distributed in 3 groups: (i) 2018 and earlier (25% of isolates), (ii) 2019 (60.9% of isolates), and (iii) 2020–2021 (14.1% of isolates). The geographic origin of the isolates and the corresponding pig production during the sampling period is depicted in Additional file 4. In particular, the isolates were recovered from farms located in 13 out of 17 Autonomous Communities in Spain (Additional file 4). Isolates from Asturias, La Rioja, Canarias Islands, and Baleares Islands, where pig production is rather low, were not included in this study. Most of the isolates were obtained from Aragón (34.6%), Catalonia (21.7%), Castilla y León (14.1%), and Andalusia (8.3%), the highest Spanish pig producers in the sample period (Additional file 4). In general, the percentage of isolates from each Autonomous Community nicely correlate with the global contribution of each Autonomous Community to the Spanish pig production (Additional file 4).

### MLST distribution

**Figure 1:**
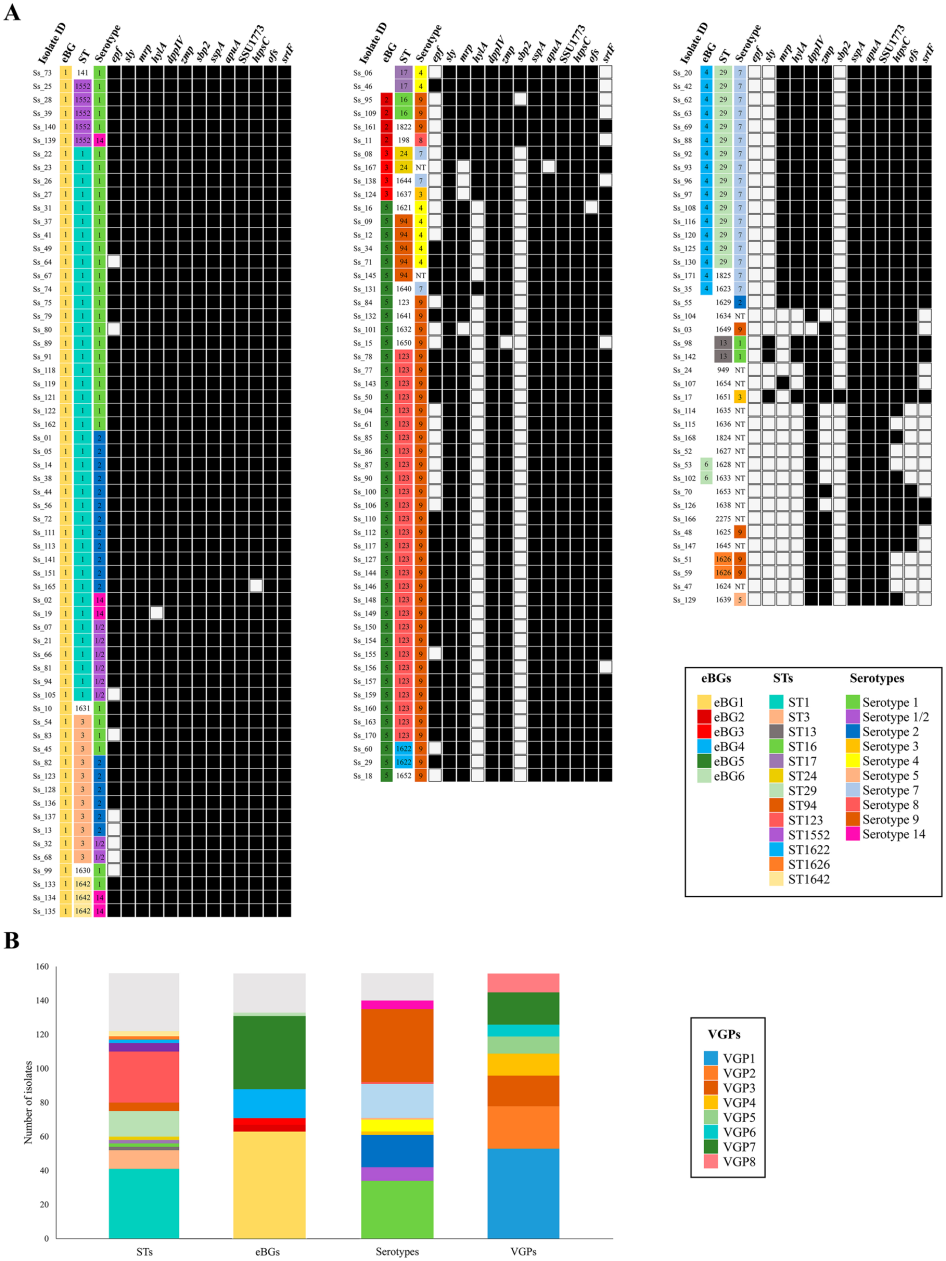
**Genetic characteristics of invasive isolates analysed in this study**. **A** Individual genetic associated profiles. The ST, eBG, and serotype are coloured as indicated in the legend. The presence (black box) and absence (white box) of 13 VGs are shown for each isolate. **B** Distribution of genotypes within the entire population. VGPs are defined in Table 1. NT refers to non-tippable isolates.

**Figure 2:**
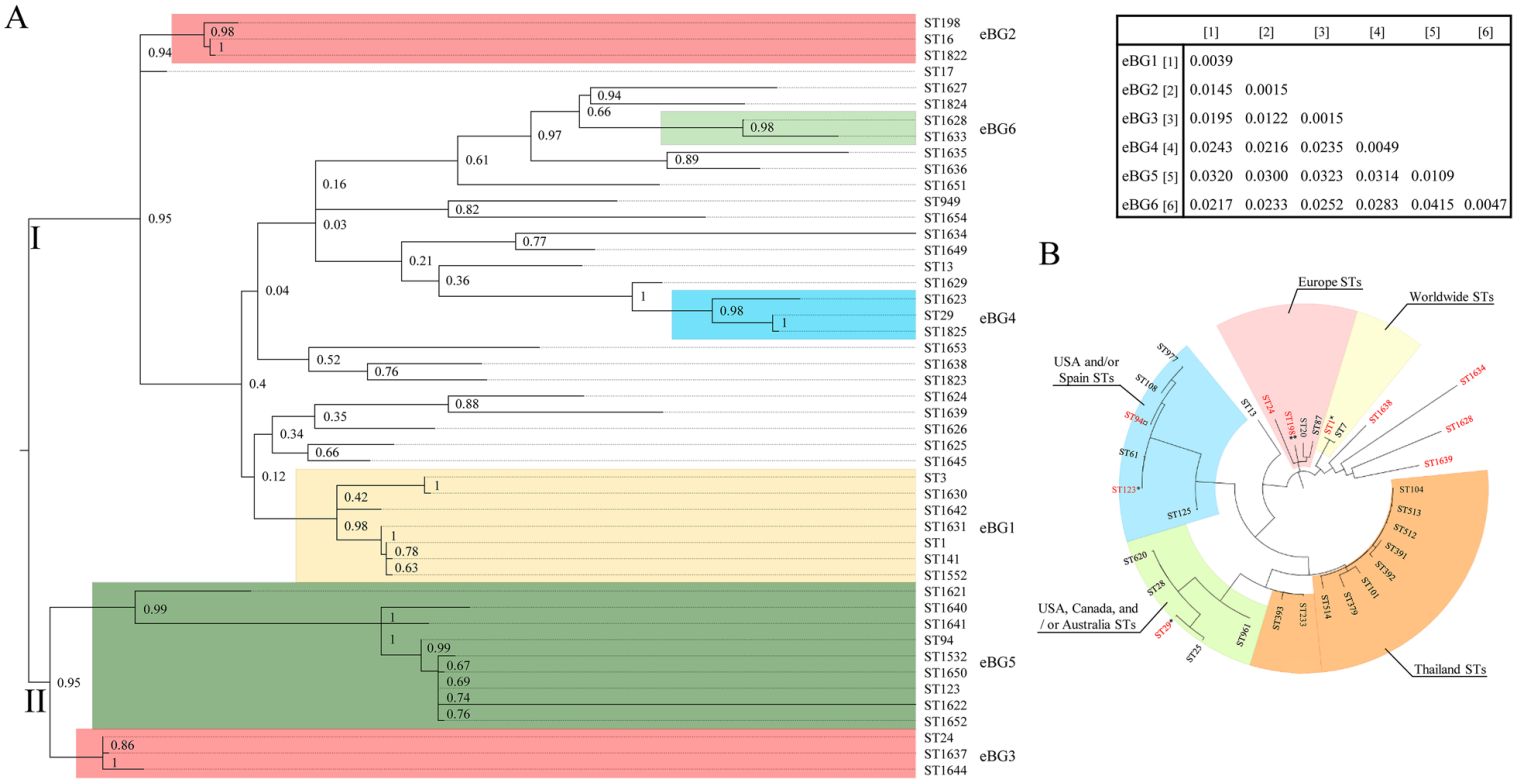
**MLST-based phylogenetic relationships**. **A** Maximum-likelihood phylogenetic tree inferred from concatenated sequences of the MLST allelic loci of all clinical isolates of this study. The bootstrap values of the internal nodes and the genetic distance within and between eBGs (inset) are shown. **B** Maximum-likelihood phylogenetic tree based on the representative STs from Spain and the major prevalent STs reported globally. STs representing results from our study in Spain are colored in red. ⁑ST found in Spain and other European countries. * ST found in Spain and other European or Asian countries. ¤ ST found in Spain and other countries of Europe, Asia and North America. ˟ ST found in Spain and worldwide.

The genetic diversity within our *S. suis* collection was analysed with MLST. An MLST scheme is available for *S. suis* using seven different house-keeping genes (*cpn60*, *dpr*, *recA*, *aroA*, *thrA*, *gki*, and *mutS*) [18]. Up to 47 different STs were identified in our collection (Figures 1A and B). Many isolates (61.6%) were assigned to 4 STs, comprising ST1 (26.3%), ST123 (18.6%), ST29 (9.6%) and ST3 (7.1%), the remaining STs included a reduced percentage of isolates (< 4%) (Figure 1B). A total of 19 new alleles for the 7 genes were identified in this study and constituted 18 novel STs. A total of 16 new STs were obtained as a result of the combination of pre-existing alleles. Altogether, 34 out of the 47 STs (24.4% of the isolates) were discovered in this work. Based on their genetic proximity, STs were grouped into CCs and eBurst groups (eBG). Up to 10 CCs were identified. Most of isolates grouped into CC1 (39.8%), CC123 (23.1%), and CC29 (10.3%). A minor group of isolates belonged to CC16 (2.6%), CC20 (1.3%), CC24 (2.6%), CC28 (1.3%), CC94 (3.9%), CC13/149 (1.3%) and CC1628/1633 (1.3%). Interestingly, CC1628/1633 was assigned here for the first time. The remaining isolates (12.8%) were unclassified in CCs. STs were also grouped in eBGs, which are widely used in classification of bacteria [46]. 28 out of 48 STs were allocated in 6 eBGs. Most of the isolates belonged to eBG1 (40.4%), eBG5 (27.6%), and eBG4 (10.9%), while eBG2 (2.6%), eBG3 (2.6%), and eBG6 (1.3%) were less represented. We found that 14.7% of the isolates were singletons (Figure 1B). Next, MLST-based phylogenetic analysis revealed two main clusters. Cluster I included three groups. The first group presented isolates of eBG2, the second group included isolates of ST17, and the third group included isolates of eBG6, eBG4, eBG1, and all singletons (Figure 2A). Cluster II was constituted by isolates of eBG5 and eBG3. To contextualize the genetic diversity of the Spanish isolates, a phylogenetic tree was constructed with the representative STs found in this work (ST1, ST13, ST94, ST198, ST123, ST24, ST1628, ST1634, ST1638, ST1639) and the major globally distributed STs from invasive disease isolates. We included worldwide distributed STs (ST1, ST28, ST94 [6, 33, 47], and ST13 [PubMLST database]), STs previously detected in Spain (ST61 [48], ST125 [10], ST24, ST123 [PubMLST database]), in several European countries (ST20 [6], ST87 [19], ST13, ST198, and ST25 [PubMLST database]), in Thailand (ST101, ST104, ST233, ST379, ST391, ST392, ST393, ST512, ST513, ST514 [6], ST620 and ST961 [PubMLST database]), in China (ST7 [6]), in USA (ST108, ST961, ST977 [6], ST25 [6, 33], ST87, and ST620 [PubMLST database]), in Canada (ST25 [6, 33] and ST620 [47]), and in Australia (ST25 [6, 33]). Five phylogenetic clusters were obtained, where a large endemic cluster contained STs only reported in Thailand. The most prevalent Spanish STs reported here were associated to 4 out of the 5 clusters (thus except the Thailand group), and novel STs clustered independently. Hence, invasive Spanish *S. suis* isolates constitute a highly variable genetic population organized in phylogenetic clusters.

### Serotype distribution

**Figure 3:**
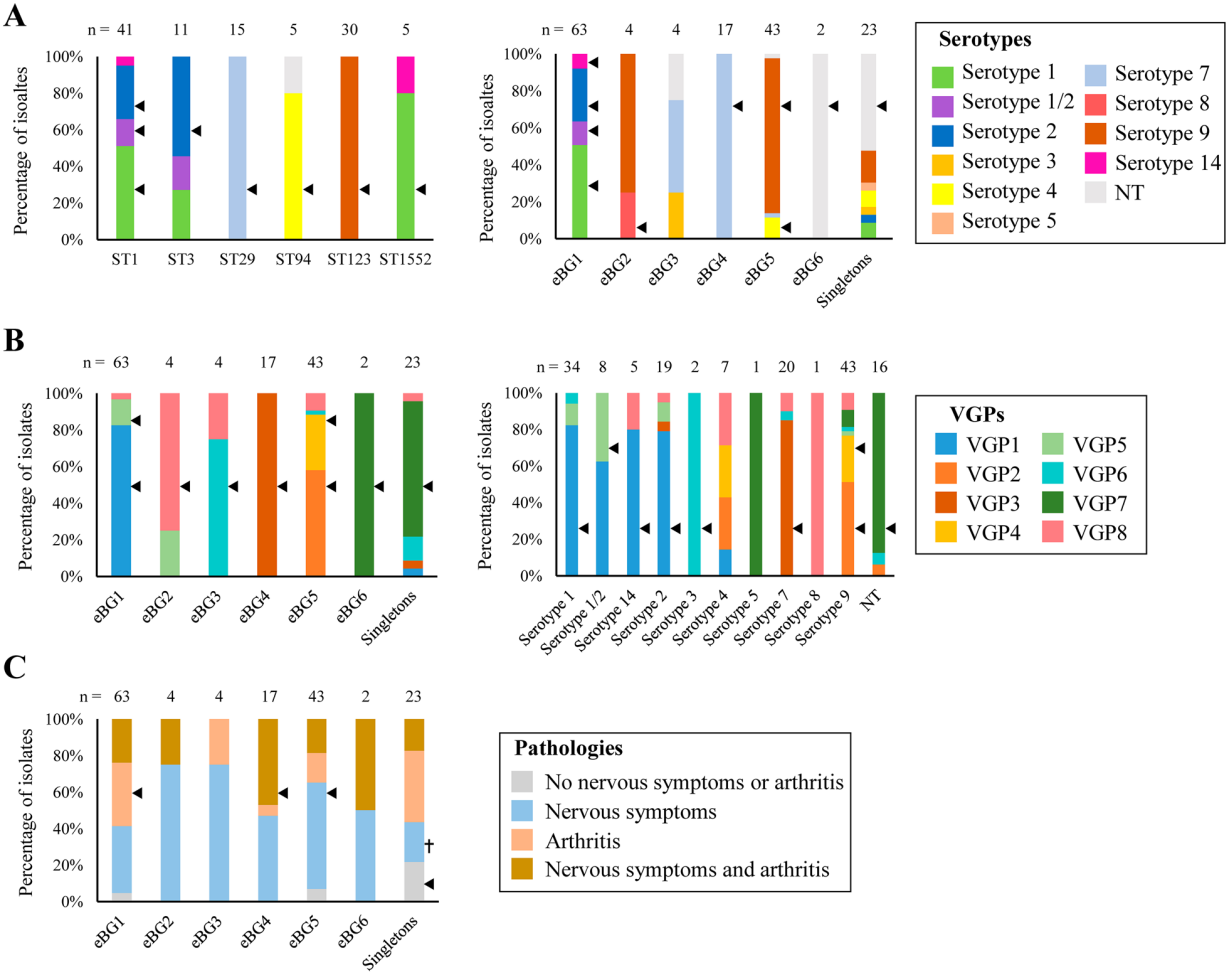
**Relationships between genotypes, serotypes, and pathotypes**. Distribution of (**A**) serotypes, (**B**) VGPs, and (**C**) pathologies within the most prevalent STs, eBGs, and serotypes. Significant associations using the Fisher’s exact test were considered at *p* < 0.05. Arrows and crosses indicate positive and negative associations, respectively.

Serotyping multiplex PCRs identified up to 10 serotypes in our collection (Figures 1A and B), 16 isolates (10.3%) were non-typable (NT). Serotypes 9 (27.6%) and 1 (21.8%) prevailed, followed by serotypes 7 (12.8%), 2 (12.2%), 1/2 (5.1%), 4 (4.5%). We identified many statistically significant associations between serotypes and STs (Figure 3A and also the statistical results are further detailed in Additional file 5). Isolates of ST1 belonged mostly to serotypes 1 with a 51.2% of the isolates (*p* < 0.001), 1/2 with a 14.6% (*p* = 0.004), and 2 with a 29.3% (*p* < 0.001). 54.0% of ST3 isolates belonged to serotype 2 (*p* < 0.001), the other half of ST3 isolates belonged to serotypes 1 (27.3%) and serotype 1/2 (18.2%), but this particular association was not significant (although these capsule types are closely related). 80% of ST1552 isolates were associated with serotype 1 (*p* = 0.008), the rest belonged to serotype 14 (*p* > 0.05). These STs belonged to eBG1 (Figure 2A), which was associated with serotypes 1, 1/2, 2, and 14 (*p* < 0.01) (Figure 3A). Only one isolate was of serotype 8 and belonged to eBG2. All the isolates of eBG4, mostly of which belonged to ST29, were associated with serotype 7 (*p* < 0.001). 83.7% of the isolates of eBG5 were associated with serotype 9 (*p* < 0.001), mainly those of ST123 (69.8%), and 11.6% were associated with serotype 4 (*p* = 0.018). 4 out 5 isolates of ST94 (11.6% of eBG5) were associated with serotype 4 (*p* < 0.001). The only two isolates of eBG6 (*p* = 0.01) and 52.2% of singletons (*p* < 0.001) were NT, and those tippable singletons were of serotype 5 (4.3%), 1 (8.7%), 1/2 (4.3%), 3 (4.3%), 4 (8.7%), and 7 (17.4%), but without statistical relationship (*p* > 0.05). Interestingly, only serotype 5 was present in singletons. In conclusion, the data revealed strong associations between some ST genotypes and capsule type.

### Distribution of VGs

The *S. suis* isolates were examined for the presence of a set of genes coding for proposed virulence factors (Figure 1A). SSU1773 (100%), *sspA* (100%), *dppIV* (99.36%), *apuA* (99.36%), and *htpsC* (95.51%) prevailed. In contrast, *sbp2* (44.23%), *epf* (51.92%), and *hylA* (60.26%) were the most variables. Based on the presence/absence of these VGs, up to 8 VG profiles (VGP) were identified (Table 1). VGP1 to VGP5 presented a conserved gene pattern in all the isolates. VGP1 was constituted by all the VGs. VGP2 to VGP5 lacked a few genes that were present in VGP1. In contrast, VGP6-VGP8 showed a conserved and variable gene pattern (Table 1). VGP1 prevailed (34%), followed by VGP2 (21.8%) and VGP7 (12.2%) (Figure 1B). Statistically significant associations between VGPs and eBGs and serotypes were detected (Figure 3B and further detailed in Additional file 5). 82.5% of the isolates of eBG1 group were significantly associated with VGP1 (*p* < 0.001) and 14.3% to VGP5 (*p* = 0.001), which included practically all VGs. However, differences occurred between the most prevalent STs. VGP1 was present in 87.8% of the isolates of ST1 (*p* < 0.001), 54.5% of isolates of ST3 (*p* = 0.186), and all isolates of ST1552 (*p* = 0.001). Three out of four isolates of eBG2 and eBG3 were associated to VGP8 (*p* < 0.001) and VGP6 (*p* < 0.001), respectively. VGP3 was present in all isolates of eBG4 (*p* < 0.001). VGP2 and VGP4 were significantly associated with 58.1% and 30.2% isolates of eBG5 (*p* < 0.001), respectively. VGP7 was present in the two isolates of eBG6 (*p* = 0.014), and in 73.9% of singletons (*p* < 0.001). In addition, significant associations were found between individual genes and phylogenetic groups. For example, *epf* (85.7%, *p* < 0.001), *zmp* (100%, *p* = 0.011), *ofs* (100%, *p* = 0.003), and *sbp2* (100%, *p* < 0.001) were statistically associated with eBG1, *sly* with eBG1 (100%, *p* < 0.001) and eBG5 (100%, *p* < 0.001), and *mrp* and *srtF* with eBG1 (100%, *p* < 0.001; and 100%, *p* < 0.001, respectively) and eBG5 (97.7%, *p* = 0.004; and 95.3%, *p* = 0.017, respectively). *hlyA* was associated with eBG1 (98.4%, *p* < 0.001) and eBG4 (100%, *p* < 0.001). Singleton isolates were negatively associated to *epf*, *sly*, *mrp*, *hylA*, *zmp*, *sbp2*, *htpsC*, *ofs*, and *srtF* (*p* ≤ 0.001). By contrast, *htpsC*, *dppIV*, SSU1773, *sspA*, and *apuA* genes were not associated with any genotype. Regarding serotypes, the two isolates of serotype 3 contain VGP6 (*p* = 0.002), while VGP1 was present in isolates of different serotypes, including serotype 1/2 (62.5%), serotype 4 (14.3%), but significantly associated with isolates of serotype 1 (82.4%, *p* < 0.001), serotype 14 (80%, *p* = 0.046), and serotype 2 (78.9%, *p* < 0.001). VGP2 was present in 28.6% of isolates of serotype 4 and significantly associated with serotype 9 (51.2%, *p* < 0.001). VGP5 contained isolates of different serotypes (1, 2, and 9), and was significantly associated with serotype 1/2 (*p* = 0.009). To summarize, *S. suis* clusters contain a fixed or variable repertoire of VGs.

**Table 1:** **VGPs detected by a PCR screening in 156 invasive *S. suis* isolates**

VGPs	Conserved gene pattern	Variable gene pattern
VGP1	*epf* + *sly* + *mrp* + *hylA* + *dppIV* + *zmp* + *sbp2* + *sspA* + *apuA* + SSU1773 + *htpsC* + *ofs* + *srtF* +	–
VGP2	*epf* + *sly* + *mrp* + *hylA*-*dppIV* + *zmp* + *sbp2*-*sspA* + *apuA* + SSU1773 + *htpsC* + *ofs* + *srtF* +	–
VGP3	*epf*-*sly*-*mrp* + *hylA* + *dppIV* + *zmp* + *sbp2*-*sspA* + *apuA* + SSU1773 + *htpsC* + *ofs* + *srtF* +	–
VGP4	*epf*-*sly* + *mrp* + *hylA*-*dppIV* + *zmp* + *sbp2*-*sspA* + *apuA* + SSU1773 + *htpsC* + *ofs* + *srtF* +	–
VGP5	*epf*-*sly* + *mrp* + *hylA* + *dppIV* + *zmp* + *sbp2* + *sspA* + *apuA* + SSU1773 + *htpsC* + *ofs* + *srtF* +	–
VGP6	*epf*-*sly* + *dppIV* + *zmp* + *sspA* + SSU1773 + *htpsC* + *ofs* +	*mrp, hylA, sbp2, apuA, srtF*
VGP7	*epf*-*sly*-*hylA*-*sbp2*-*sspA* + *apuA* + SSU1773 +	*mrp, dppIV, zmp, htpsC, ofs, srtF*
VGP8	*sly* + *mrp* + *dppIV* + *sspA* + *apuA* + SSU1773 +	*epf, hylA, zmp, sbp2, htpsC, ofs, srtF*

The symbols + and – refer to gene presence and absence, respectively. Note that VGPs from 1 to 5 show a conserved gene pattern, while VGPs from 6 to 8 show conserved and variable gene patterns.

### Relationship between genetic lineages, pathotyping, and origin of isolates

Firstly, we analysed the relation between pathotypes and the genetic origin of the isolates. A summary of the results is illustrated in Figure 3C and further detailed in Additional file 5. 95.2% of the isolates of eBG1, mainly those of ST3, affected either the nervous system and/or arthritis, mostly separately (71.4%, *p* = 0.029) than in combination (23.8%). About half of isolates of eBG4 (47.1%) were recovered from cases with nervous alterations and arthritis (*p* = 0.03), but it was not associated with only nervous alterations (47.1%, *p* = 0.76) or only arthritis (5.9%, *p* = 0.73). In contrast, more than half (58.1%) of the isolates of eBG5 caused only nervous alterations (*p* = 0.024). Finally, singletons were negatively associated with alterations in the nervous system (21.7%, *p* = 0.022). Besides, 81.8% of the VGP8-containing isolates only produced nervous symptoms (*p* = 0.011), and VGP3-containing isolates produced either nervous system and/or arthritis (94.4%), mostly both pathologies (44.4%, *p* = 0.039). Thus, several genetic lineages with particular virulence traits are related to host-associated pathologies.

Secondly, we analysed the statistical associations between the anatomical site of isolation and the genotype of the isolates. Only isolates recovered from a unique organ were taken into account, the rest were considered as non-defined. 47.8% of singletons (*p* = 0.024) and 47.4% of isolates of VGP7 (*p* = 0.048), were recovered from joints. 90.9% of isolates of VGP8 (*p* = 0.011) and 72.11% of isolates of eBG5 (*p* = 0.005) were recovered from CNS, mostly the ones of ST123 and serotype 9. We did not find statistically significant associations between the genetic origin of the isolates and those recovered from nasal turbinate, probably due to the low number of isolates recovered from this site. Concerning the age of the host, streptococcal disease in suckling piglets was significantly caused by isolates of serotype 1/2 (50%, *p* = 0.002), and NT isolates (37.5%, *p* = 0.001) but not significant associations were found between the genetic origin of the isolates with transition or fattening pigs. Concerning the year of isolation, we found that isolates of VGP7 were mostly recovered in 2018 or earlier (47.4%, *p* = 0.024) compared to 2019 (36.8%, *p* = 0.022) and, isolates of serotype 1/2 were mostly recovered from 2020 (50%, *p* = 0.014) compared to 2019 (25%, *p* = 0.057).

### Identification and analysis of core genome

**Figure 4:**
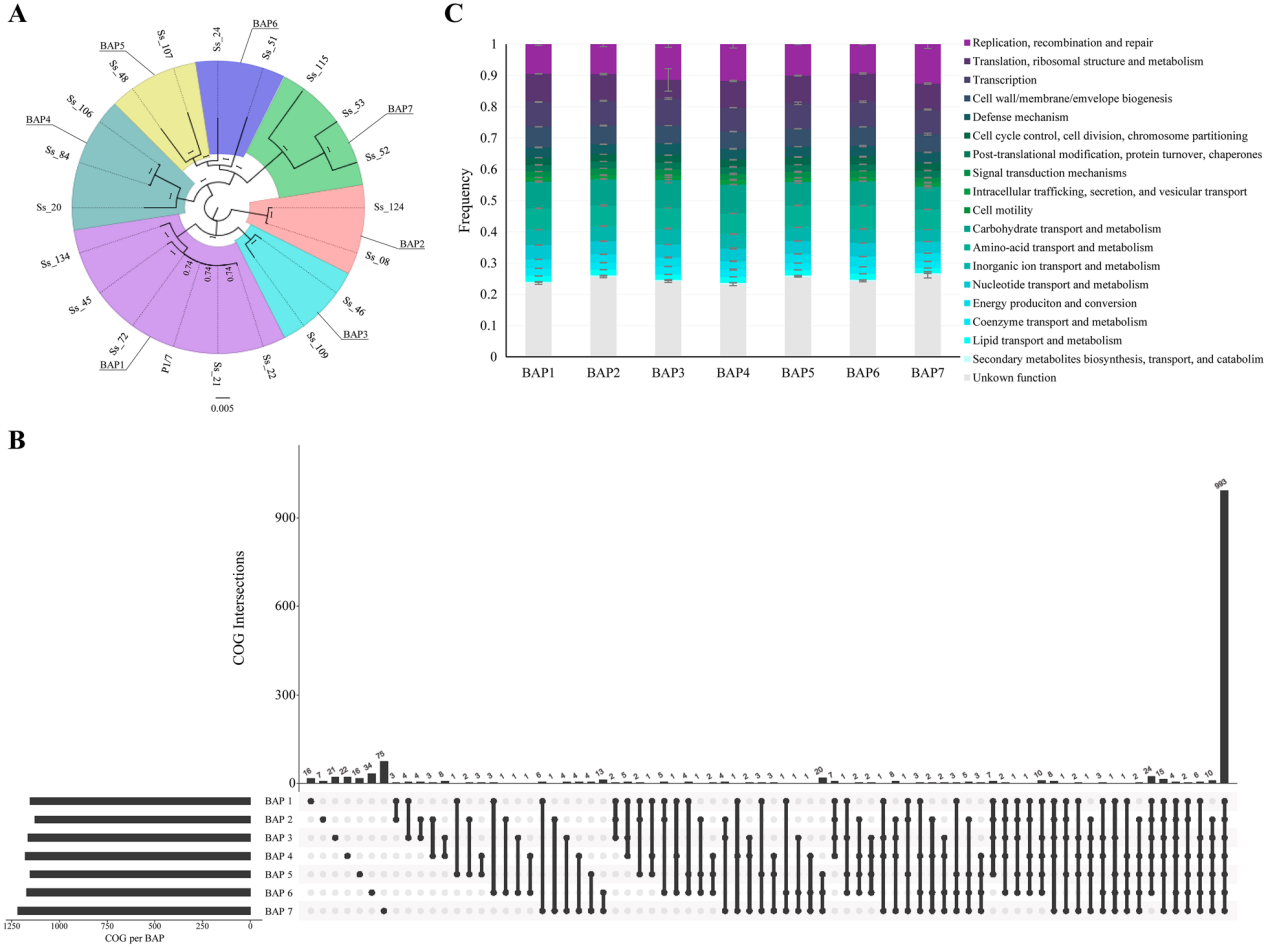
**Pan-genome analyses**. **A** Phylogenetic tree of the core genome distributed in seven BAP groups. **B** Number of shared and unshared genes among BAPs. **C** Distribution of Gene Ontology Categories (COGs) present in BAPs. Error bars indicate standard variation.

We then expanded the knowledge on the genetic variability of Spanish isolates by sequencing the entire genome of a set of isolates of different STs. Selected isolates were three of ST1, two of ST123, and one of ST3, ST24, ST1637, ST3, ST1642, ST16, ST29, ST1628, ST949, ST17, ST1625, ST1626, ST1627, ST1654, and ST1637. Hence, these isolates represented the genetic diversity established by MLST clustering (Figure 2A). The number of coding sequences (CDs) of the isolates ranged from 1915 to 2503. The core genome was constituted of 1049 CDs, which represents about 41–54% of the entire genome. The genetic structure of the population was determined by a Bayesian clustering method and produced 7 BAP groups. The phylogenetic tree analysis evidenced that the tree BAPs can split into two clades: BAPs 1 to 3 and BAPs 4 to 7 (Figure 4A). Interestingly, BAP1 contained isolates of ST1 (Ss_22, Ss_21, Ss72), ST3 (Ss_45), and ST1642 (Ss_134), all of them of eBG1. The reference strain P1/7 was also included in this group (Figure 4A). BAP2 was composed of two isolates (Ss_124 and Ss_08), both of them of eBG3. BAP3 was composed of an isolate of eBG2 (Ss_46) and a singleton (Ss_109) which was genetically related to eBG2 (Figure 2A). BAP4 was constituted of three isolates, one of eBG4 (Ss_20) and two of eBG5 (Ss_84 and Ss_106). BAP5 included two isolates (Ss_48 and Ss_107) and both were genetically related singletons (Figure 2A). BAP6 included two isolates (Ss_24 and Ss_51) both of them closely related singletons (Figure 2A). Finally, BAP7 was constituted of three isolates, Ss_115 of eBG3, Ss_53 of eBG6 and Ss_52 that is a singleton closely associated with eBG6 (Figure 2A). To conclude, in general, the genetic structure determined by the BAPs groups matched with MLST, except for some isolates of BAP4 and BAP7.

The number of shared and unshared genes within BAPs groups is summarized in Figure 4B. Shortly, the number of shared genes within BAPs was 993, while the number of shared genes between certain BAPs ranged from 24 to 3. Besides, the number of unique genes for each BAP ranged from 75 (BAP7) to 16 (BAP5). These findings evidence that BAP7 has a larger dispensable genome than other BAPs. According to the Gene Ontology Categories (COG), most of the genes belonged to 18 COGs, and a considerable amount of them were associated with *i*) information, storage, and processing (23% of the genome), *ii*) cellular processes, and signalling (15% of the entire genome), and *iii*) metabolism (8% of the entire genome). Hypothetical genes with unknown functions constituted about 25% of the genome. Figure 4C shows the COG distribution for each BAP group. Statistical comparisons within BAPs evidenced a significant set of genes over and under-represented for each BAP (Additional file 6). Briefly, BAP7 exhibited the largest number of over (*n* = 117) and under (*n* = 129) represented genes compared to the other BAPs. Many over-represented genes are involved in replication, recombination, cell division, and repair systems such as crossover junction endodeoxyribonuclease, DNA excision and repair, nucleases, DNA synthesis, endonucleases, and recombinases, among others. Over-represented genes in BAP7 coded for transposases, phage integrases, and putative proteins related to cell envelope synthesis including components of SecA2 machinery, capsule biosynthesis, sialidases, exo-DNases, proteases (i.e., Zmp protease), and unknown putative exoproteins. In contrast, a large number of genes (*n* = 129) were underrepresented or just lacked in BAP7. For example, genes coded for hypothetical nutrient uptake systems, metabolic enzymes, response regulators, and restriction-modification systems, among others. These data revealed that isolates of BAP7 have very different virulence and metabolic genetic profiles compared to other BAPs. BAP1 included a substantial number of over (*n* = 19) and under (*n* = 26) represented genes that belong to different categories, including membrane transporters, metabolic enzymes, and capsule biosynthesis. BAP2 to BAP6 included a minor number of over and under-represented genes that varied from 1 to 8 and were involved in different functions such as DNA replication, nutrient uptake, putative proteases and metabolic enzymes, regulators, and secreted proteins. In addition, we inspected the genomes to identify the presence/absence of 30 novel VGs and MGEs. Most of the VGs were found in all the genomes (Additional file 7), but the genes *cbp40*, *fhb*, *fhbp*, *ltaA*, *neuB*, and *tran* were only present in 7, 16, 18, 15, 7, and 9 genomes, respectively. Interestingly, isolates of eBG1 (BAP1) presented all the virulent genes (ASR = 4.4,* p* = 0.001) and showed that this is a highly invasive clonal group, in agreement with our PCR screenings (Figure 1A). All isolates of eBG3 (BAP2) and eBG4 lacked *cbp40*, *neuB,* and *tran*, while *cbp40* and *neuB* were poorly observed in isolates of eBG5 (ASR = 4.4,* p* = 0.047). Most singletons lacked several genes. A total of 378 MGEs were found, comprising 8 insertion sequences, 6 transposons, and 1 integrative and conjugative element (Additional file 8). The most prevalent insertion sequences were IS*Ssu7*, IS*Ssu13* and IS*Ssu6* (27.0%, 15.1%, and 10.1%, respectively). Composite transposons were identified, including 26 of cn_IS*Ssu7*, 20 of cn_IS*Ssu6*, and 19 of cn_IS*Ssu13*. In addition, genes from plasmids *rep22* and *repUS43* were identified. All the isolates carried MGEs, but its number ranged from 43 to 4. The VGs analysed in this study were not present in MGEs.

### Genetic arrangements in particular VGs

**Figure 5:**
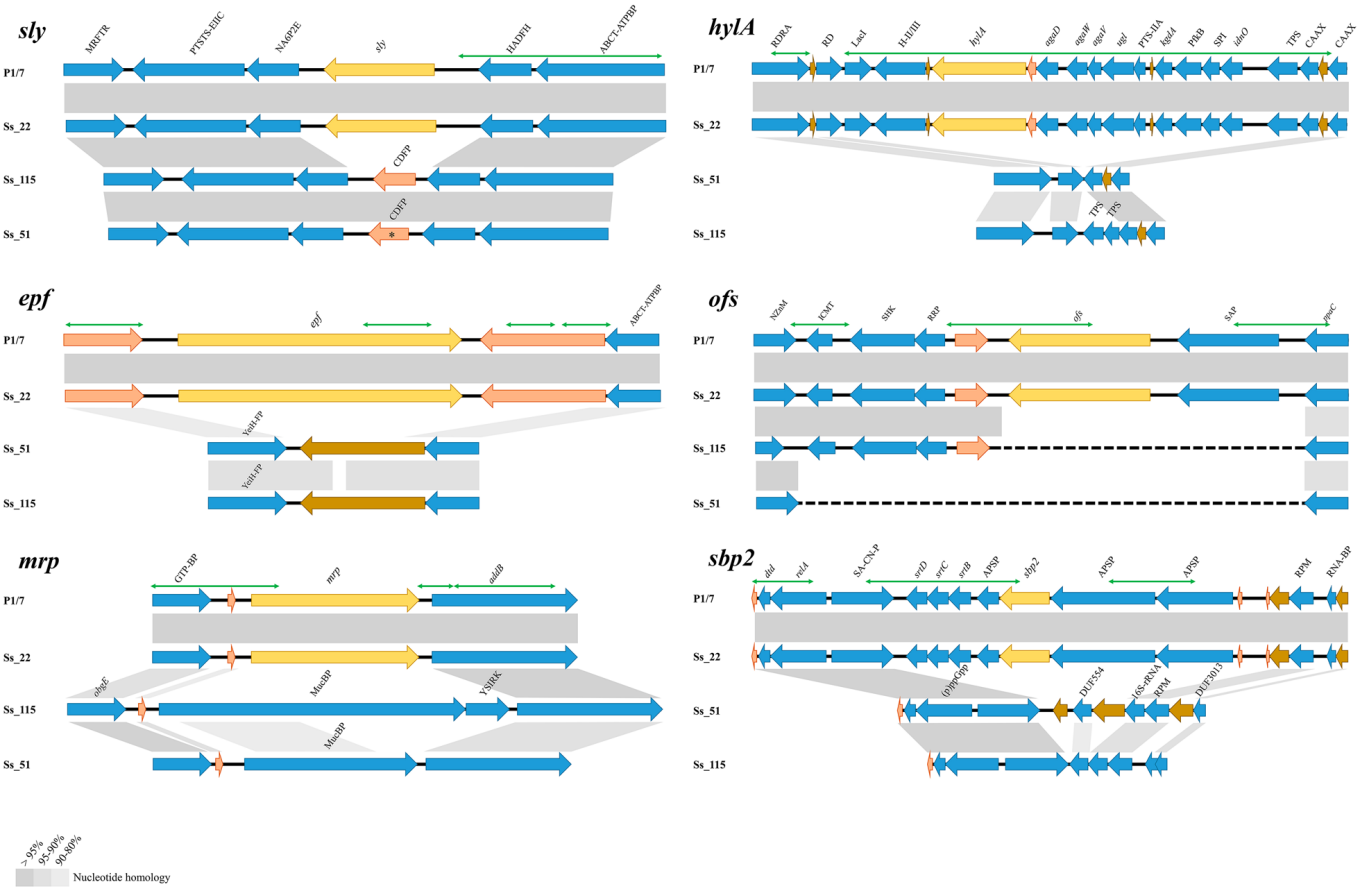
**Organization of VGs in *S. suis* genomes**. The genes *epf*, *sly*, *mrp*, *hylA*, *ofs* and *sbp2* (yellow arrows) in the genome P1/7 and the isolates Ss_20, Ss_51 and Ss_115 are shown. The flanking genes are blue-coloured. Genes coding for putative membrane proteins and hypothetical proteins are shown in orange and dark olive-coloured, respectively. Regions sharing more than 80% of sequence similarity are indicated with grey shadows. The regions in which recombination breakpoints were estimated are shown with green arrows (above). MRFTR: MurR/RpiR family transcriptional regulator; PTSTS-EIIC: PTS transporter subunit EIIC; NA6P2E: N-acetylmannosamine-6-phosphate 2-epimerase; sly: Suilysin (hemolysin); HADFH: HAD famlily hydrolase; ABCT-ATPBP: ABC transporter ATP-binding membrane protein; CDFP: Carboxymuconolactone decarboxylase family protein; epf: Putative surface-anchored protein; YeiH-FP: YeiH family protein; GTP-BP: GTP-binding protein; mrp: Muramidase-released protein precursor; addB: Putative ATP-dependent exonuclease; obgE: GTPase ObgE; MucBP: MucBP domain-containing protein; YSIRK: YSIRK-type signal peptide-containing protein; RDRA: Ribonucleoside-diphosphate reductase alpha; RD: Ribonucleoside-diphosphate; LacI: LacI family regulatory protein; H-II/III: Heparinase II/III-like protein; hylA: Hyaluronidase precursor; agaD: putative N-acetylgalactosamine-specific phosphotransferase system (PTS), IID component; agaW: putative N-acetylgalactosamine-specific phosphotransferase system (PTS), IID component; agaV: putative N-acetylgalactosamine-specific phosphotransferase system (PTS), IID component; ugl: Putative unsaturated glucuronyl; PTS-IIA: Putative N-acetylgalactosamine-specific phosphotransferase system (PTS), IIA component; kgdA: putative KHG/KDPG aldolase; PfkB: PfkB family carbohydrate kinase; SPI: Putative sugar-phosphate isomerase; idnO: Putative gluconate 5-dehydrogenase; TPS: Transposase; CAAX: CAAX amino terminal protease family membrane protein; NZnM: Putative neutral zinc metallopeptidase; ICMT: Isoprenylcysteine carboxyl methyltransferase family protein; SHK: Sensor histidine kinase; RRP: Response regulator protein; ofs: Serum opacity factor; SAP: Putative surface-anchored protein; ppaC: Manganese-dependent inorganic; dtd: D-tyrosyl-tRNA(Tyr) deacylasE; relA: GTP pyrophosphokinase; SA-CN-P: Putative surface-anchored 2',3'-cyclic-nucleotide 2'-phosphodiesterase; srtD: Sortase SrtD; srtC: Sortase SrtC; srtB: Sortase SrtB; APSP: Putative accessory pilus subunit protein; sbp2: Major pilus subunit protein; RPM: 50S Ribosomal protein L11 methyltransferase; RNA-BP: Putative RNA-binding protein; (p)ppGpp: (p)ppGpp syynthetase/guanosine-3’,5’-bis(diphosphate 3’-pyrophophohydrolase; DUF554: DUF554 domain containing protein; 16S-rRNA: 16S rRNA (uracil(1498)-N(3))-methyltransferase; DUF3013: DUF3013 family protein.

We hypothesized that the variable composition of certain VGs in particular genetic lineages could have originated from recombination events. Therefore, we studied the genetic context of *epf*, *sly*, *mrp*, *hylA*, *ofs*, *sbp2*, *htpsC*, *srtF*, and *zmp*, which showed a low prevalence within our *S. suis* population (Figure 5). In some genomes, *epf*, *sly* and *mrp* were replaced by genes coding for two hypothetical membrane proteins. Recombination breakpoints were predicted in both flanking regions (Additional file 9), but only located upstream of the *sly* gene. In contrast, *hylA*, *ofs,* and *sbp2* revealed large genomic rearrangements in some isolates that affected flanking regions with several genes. Many recombination breakpoints were detected along these sequences (Additional file 9). Thus, the variability of the cited VGs is favoured by extensive recombination, which also produced a concomitant loss or gain of flanking genes. To investigate the strength of selection in these VGs, the dN/dS ratio was estimated by maximum likelihood (Table 2). Note that a dN/dS estimate around 1 is usually interpreted as a genetic signature of neutral evolution, lower than 1 is interpreted as negative selection for amino acid replacement (purifying selection), and higher than 1 is interpreted as positive selection for amino acid replacement (diversifying selection) [49]. The estimated dN/dS for most of the genes ranged from 0.05 to 0.49, indicative of a strong negative selection. The estimated dN/dS for *sspA* was 0.82 while for *ofs* it was 2.35 indicating genetic signatures of strong positive selection. Altogether many VGs are prone to extensive genetic exchange but retain a high level of amino acid conservation, probably to maintain fundamental properties of the protein such as function and stability. An extreme case was *sbp2*, which did not present any mutation. In contrast, as previously indicated the gene *ofs*, which is also prone to recombination, revealed genetic signatures of diversifying selection.

**Table 2:** **Estimated selection pressures for the studied VGs in 19 *S. suis* isolates.**

Gene	Estimated dN/dS	Number of codon sites with significant selection pressure
*epf*	0.33 (0.27–0.40)	–
*sly*	0.05 (0.00–0.25)	–
*mrp*	0.33 (0.30–0.37)	9 NSSs
*hylA*	0.49 (0.40–0.60)	1 NSSs
*htpsC*	0.32 (0.22–0.46)	2 NSSs
*zmp*	0.24 (0.23–0.26)	64 NSSs
*ofs*	2.35 (2.17–2.54)	1 NSSs
*sbp2*	–	–
*strF*	0.33 (0.08–0.87)	–
*sspA*	0.82 (0.73–0.92)	12 NSSs
SSU1773	0.24 (0.22–0.27)	50 NSSs
*apuA*	0.10 (0.09–0.12)	130 NSSs
*dppIV*	0.15 (0.13–0.17)	52 NSSs

The table shows the estimated global dN/dS (including the 95% confidence interval) and the number of negatively selected codon sites (NSSs) at a significance level of 0.05. Positively selected sites at that significance level were not found. The gene *sbp2* was totally conserved.

## Discussion

Spain is nowadays the leading pig producer in Europe. Here we found that ST1 (26.3%) prevailed within invasive isolates followed by ST123 (18.6%), ST29 (9.6%), and ST3 (7.1%). This finding was not surprising because these STs were reported in different studies performed in other European countries. For example, of 124 *S. suis* isolates recovered in the Netherlands between 1996 and 2008, ST16 (42.7%) prevailed, followed by ST1 (22.6%) and ST29 (4.8%) [50]. Another study analysing 18 isolates in Germany, obtained between 1996–1998 and between 2001–2004, belonged to ST1 (21.1%), ST28 (10.5%), ST29 (10.5%), and ST98 (10.5%) [19]. In 78 *S. suis* isolates obtained in Italy between 2017 and 2019 ST1 (21.8%) and ST123 (21.8%) prevailed, followed by ST7 (11.5%), ST1547 (9%), ST29 (7.7%), ST16, ST94, and ST1540 (3.8% each) [51]. In United Kingdom, the most prevalent STs in 116 *S. suis* isolates recovered between 2002–2013 were ST1 (49.1%), ST29 (6.9%), ST25 (6%), ST2 (4.3%), and ST28 (4.3%) [1]. The PubMLST database indicates 47 strains isolated in France between 1995–2019 that belonged to ST1 (16%), ST81 (4%) and ST140 (4%). In conclusion, considering current data, *S. suis* strains of ST1, ST123 and ST29 are highly invasive. Also, these data suggest variations in the distribution of these STs among European countries.

Our findings are in partial agreement with early epidemiological studies in Spain, which revealed that ST1 was the most prevalent among invasive isolates [1, 10]. Indeed, ST1 isolates harbour an abundant and conserved repertoire of VGs and genes coding for capsule 2. This is consistent with previous reports in different countries [51], and may explain its high prevalence. Yet, our work revealed differences from previous work in Spain [1, 10]; first, isolates of ST1 represented about 26% of our collection, which is a considerable reduction compared to previous studies in where ST1 represented about 50% of the isolates [1], while the prevalence of ST123 that we observed was similar to previous work. Second, we identified up to 48 STs of which 44 were not previously reported. Unfortunately, only one old study characterized *S. suis* by MLST [1]. But, a comparison of several previous serotyping-based studies performed in Spain also revealed substantial differences. An early study [9] reported a prevalence of serotype 2 (53%), followed by serotype 1 (9%), 1/2 (7%), and 8 (4%). A subsequent work reported a high prevalence of serotype 9 (64%) in isolates recovered between 1998–2002, followed by serotype 2 (15%), 7, 8 and 3 [8]. Recently (2021), it was reported that the most prevalent serotypes were 2 (21%), 1 (21%), 9 (19%), 3 (6%) and 7 (3%) [7]. Our serotyping studies partially agree with the latter with relative variations in some serotypes, including serotype 9 (27%), 2 (12%), and 7 (12%). These differences may be due to the different sampling strategies used in both studies. In the present study we used a representative number of *S. suis* isolates from each Autonomous Communities to represent the Spanish diversity taking into consideration the pig production. Besides, we included only one isolate per pig and farm to avoid opportunistic clones and overrepresentation of endemic clones in particular farms, respectively. All these criteria were not considered in [7] or other previous studies [8, 48]. Anyhow, the large differences with previous works [8, 48] suggest a trend over time to reduce the prevalence of serotypes 2 and 9 and increase the prevalence of other serotypes (e.g., serotype 1). Thus, these data suggest the occurrence of temporal variations in the population structure of Spanish *S. suis* and an increase in its genetic variability in recent years. In line with this suggestion we observed statistical variations of the prevalence of serotype 1/2 during a very short period of time. In a previous study with data from Germany analysing a population of 711 invasive *S. suis* isolates obtained in two different periods of time (1996–2004 and 2015–2016) [52], authors observed that serotypes 1 and 14, and 2 and 1/2 were less prevalent between 2015–2016 than between 1996–2004, while isolates of serotypes 4 and 7 were more prevalent in 2015–2016 than in 1996–2004. Also, a study performed in France based on 200 *S. suis* isolates obtained before and after 2010 revealed that serotypes 1, 2, 3, 7 and 9 were more prevalent before than after 2010, in contrast to serotype 1/2 [53]. Altogether, studies in different countries suggest that the population structure of *S. suis* is dynamic and indicate the need of regular surveillance studies to better fit vaccine design and diagnostic markers.

The present study highlights that the structure of the Spanish *S. suis* population is split into genetic clusters of high variable diversity. Certain clusters are highly conserved in terms of gene content, including VGs. An example is ST1. Phylogenetic analysis of ST1 isolates evidenced a high level of sequence similarity (BAP1) and reduced variations in gene content, despite belonging to different geographic locations. The high level of genetic conservation may be related to the success in causing infection. In contrast, other genetic clusters are more heterogeneous (i.e., eBG3, eBG5, eBG2, together with a large abundance of singletons related to eBG6 and eBG4). Interestingly, some of these isolates belong to BAP7, which was enriched in genes involved in DNA recombination and repair but lacked genes coding for several restriction-modification systems, thus suggesting that these isolates are prone to extensive genomic exchange. Hence, clusters with high genomic diversity may guarantee a rapid response to fluctuating environments. In addition to the genetic variability of Spanish *S. suis*, pan-genome analysis evidenced a core genome that accounts for ~ 47.5% of any single genome, in relative agreement with previous reports for different geographic areas [54, 55], but much lower than other studies with different streptococci such as *Streptococcus pneumoniae* (70%) [56] or *Streptococcus agalactiae* (80%) [57].

The genomic diversity of *S. suis* in Spain could be generated by several routes. First, various STs were produced by combinations of pre-existing alleles (35%) since we detected evidence of genetic exchange within prevalent lineages. In this concern, previous work on *S. suis* populations isolated in Asia and England also revealed traits of recombination [54]. Yet, many new STs were phylogenetically grouped or related to known STs (58%) (cluster I), suggesting that some new STs evolved from a common ancestor. However, a considerable percentage (37%) of ST alleles were identified for the first time in this study, and some of them were distant from the pre-established STs, indicating the emergence of new lineages. Then, did the genetics of invasive *S. suis* vary in the last decade in Spain? Since 2010, Spanish pig production increased drastically while the number of farms decreased. Factors associated with this change include increased import/export of piglets from other European countries. According to the Food of Agriculture Organization of the United Nations (FAO), in the last 3 years (2019–2021) Spain imported an average of 2,480,560 living pigs/year and exported an average of 1 596 467 living pigs/year, which involves 7.8% and 5.0% of the total of the Spanish swine production, respectively. This movement of pigs could be related to the emergence of the ST123 in Italy [51], which was only previously reported in Spain [1, 10]. Interestingly, high rates of multi-resistance to antibiotics were found in isolates of serotype 9 [7]. Also, vaccination with serotypes 2 and 1/2 can be effective, but less for serotype 9 [4]. Thus, extensive use of antibiotics and vaccines may have reduced ST1 isolates, maintained ST123 (serotype 9), and enhanced the emergence of novel STs associated with other serotypes (e.g., ST29 and singletons).

A key finding in our study was the evidence of multiple associations between genetic lineages, VG content, capsule types, and pathotypes. This suggests a multifactorial nature of *S. suis* pathogenicity. Indeed, 5 out of 13 VGs were present in all the phylogenetic clusters. Two of them, SSU1773 and *sspA*, code for surface-associated putative proteases of the subtilisin-like family. SSU1773 was identified as relevant for adherence to host cells in a screening of TraDIS libraries of *S. suis* strain P1/7 [28] and upregulated in bacteria recovered from blood, and inner tissues of experimentally infected pigs [12]. SspA-1 is secreted, and a *sspA* mutant showed attenuated virulence in mice and pig infection models [25, 26] and an impaired capacity to activate the host inflammatory response [25]. In agreement with our results, the *sspA* gene was previously amplified by PCR in 29 out of 33 *S. suis* reference strains [25]. Conversely, a study in Australia showed that *sspA* is barely present in *S. suis* isolates, even in ST1 isolates [33]. However, *S. suis* genomes of ST1 contain *sspA* gene, for example P1/7 (Accession AM946016.1), S10 (Accession LR738721.1), or GZ1 (Accession CP000837.1). Probably Australian isolates derived from a lacking-*sspA* gene ancestor. Concerning the remaining three genes, *dppIV* codes for a Dipeptidyl peptidase that degrades the antimicrobial peptides PR-39 and IL-8 [22]. The gene *apuA* encodes an Amylopullulanase that cleaves α-1-4 glycosidic bonds between glucose residues in starch and glycogen [27] and its expression is upregulated when bacteria were recovered from different inner tissues of *S. suis-*infected pigs [12]. Finally, *htpsC* encodes a histidine triad protein that mediates the adherence to extracellular matrix proteins [29] and contributes to bacterial invasion [30]. Thus, the high prevalence of these genes in our invasive isolates can be explained by a relevant role in nutrient acquisition or immune escape activities. Conversely, the *S. suis* population showed a significant variation in particular VGs such as *epf*, *sly*, *mrp*, *ofs*, *hylA*, and *sbp2*, which were also previously reported as important virulence factors [21, 34, 58]. Considering their absence in many disease-causing isolates, one may speculate if they are indeed critical for in vivo infection. Our genetic analysis evidenced major chromosomal rearrangements between *S. suis* isolates comprising these genes and their flanking regions, and thus they are likely prone to extensive genetic exchange. An advantage of the gain and loss of these genes is the possible evasion of the host immune system, while its function in virulence could be compensated by a redundant function of other factors [59]. However, most of the genes globally evolved under purifying selection, with the only exception of the gene *ofs* that revealed genetic signatures of diversifying selection. These observed purifying selection constraints could be a consequence of the required maintenance of protein stability and activity. In the case of *ofs*, the detected diversifying selection could be caused by improvements of activity and adaptation to the host immune system. This may be related to observed allelic variation [60]. Altogether, these accessory VGs that migrate between genetic lineages could be important for certain clinical outcomes, as suggested by the significant associations found in the present study. Considering that the understanding of genetic diversity is the basis for vaccinology approaches and control actions, this study contributes to increasing the knowledge of the epidemiology of Spanish *S. suis*, and it could be useful for designing strategies to control and diagnose streptococcal swine disease in one of the countries of Europe with highest pig production.

This study provides a comprehensive analysis of the genetic structure of invasive isolates of *S. suis* recovered in different geographic regions of Spain from 2014 to 2021. We conclude that a large number of genotypes can cause *S. suis* disease, but only few genotypes are responsible of most clinical cases, including ST1 (serotypes 2, 1, 1/2, and 14), ST123 (serotype 9), ST29 (serotype 7), and ST3 (serotypes 1, 1/2, and 2). These genotypes belong to three genetic clusters that are highly variable in terms of gene content and function, and include certain VGPs. As a consequence, isolates of these genotypes cause different clinical outcomes, and can target different host organs and ages. The origin of this variability seems to be caused, and controlled, by genetic exchange. Considering that the understanding of genetic diversity is the basis for vaccinology approaches and control actions, regular surveillance studies are required for monitoring the emergence of possible new variants of *S. suis*, and understanding their genomic architecture, in one of the highest pig producer countries of Europe.

### Supplementary Information

**Additional file 1:** **Clinical isolates of *S. suis* analysed in this study.**

**Additional file 2: Distribution of *S. suis* isolates based on host factors.** (A) Percentage of isolates recovered from different anatomical sites. The anatomical site of isolation of 17.3% of the isolates was not defined (ND). (B) Distribution of isolates based on the reported clinical symptoms from pigs where several pigs presented more than one symptom. (C) Percentage of isolates recovered from pigs at different production stages (suckling piglets, transition, and fattening). The age of the pigs was not reported for 26.5% of the isolates. CNS: Central Nervous System.

**Additional file 3: Primers and PCR conditions used in this study.**

**Additional file 4: Geographic origin of *S. suis* isolates**. The geographic origin of the *S. suis* isolates (white bars) for each Autonomous Communities (CCAA) and its pig production (black bars, where error bars correspond to standard deviation) according to official reports (MAPA) in the sampling period (2015-2021).

**Additional file 5: Results obtained from statistical analyses using Fisher’s exact test**. Frequency, percentage, Adjusted Standardized Residues (ASR) and *p* value are included for each association.

**Additional file 6:** **Over and under-represented genes for each BAP group**.

**Additional file 7: Presence of VGs in genomes of 19 *S. suis* isolates.**

**Additional file 8: Identification of mobile genetic elements in genomes of 19 *S. suis* isolates.**

**Additional file 9: Recombination breakpoints found in genome sequences of 19 *S. suis* isolates.**

## Data Availability

The genome sequences produced in this study are available from NCBI database (BioProject accession number PRJNA1037513).

## Abbreviations

MLST: Multi locus sequence type
ST: sequence type
THB: Todd-Hewitt Broth
THA: Todd-Hewitt Agar
OD: optical density
VG: virulence-associated gene
LRT: likelihood ratio test
CC: clonal complex
bp: base pair
dN/dS: non-synonymous/synonymous substitution
BAP: Bayesian group
MGE: mobile genomic element
ASR: adjusted standardised residues
CNS: central nervous system
eBG: EBurst group
NT: non-typpable
VGP: virulence-associated gene profile
CDs: coding sequence
COG: Gene Ontology Categories
FAO: Food of Agriculture Organization of the United Nations
