# Supplementary material for: Genomic and phenotypic analysis of invasive Streptococcus suis isolated in Spain reveals genetic diversification and associated virulence traits
Source: Vet Res. 2024 Jan 24;55:11. doi: 10.1186/s13567-024-01267-0 (PMC10807230; doi:10.1186/s13567-024-01267-0)
Supplement: Supplementary file 3 — Additional file 3: Primers and PCR conditions used in this study. [file 13567_2024_1267_MOESM3_ESM.docx]

**Additional file 3. Primers and PCR conditions used in this study.** The target gene, primer name and sequence used for *S. suis* identification, serotype, MLST and virulent associated gene detection are indicated. For each primer pairs, the annealing temperature (aT) used in the PCR, and the expected size for the PCR product are indicated.

| **Gene** | **Gene product** | **Primer** | **Sequence (3'-5')** | **aT** | **Expected PCR product (bp)** | **Reference** |
| --- | --- | --- | --- | --- | --- | --- |
| ***Streptococcus suis* identification** | | |  |  |  |  |
| *gdh* | glutamate dehydrogenase | JP4-Fw | GCAGCGTATTCTGTCAAACG | 55 | 688 | [13] |
|  |  | JP5-Rev | CCATGGACAGATAAAGATGG |  |  |  |
| *recN* | DNA repair protein RecN | recN-Fw | CTTAAAGACCGTTATCAGACAACCT | 60 | 723 | [15] |
|  |  | recN-Rev | ATCGAACTTGGAAACGAGCTTTCTC |  |  |  |
| **Capsular serotyping** | | | |  | | |
| *cps*2I-1/2I | cps2I and cps1/2I | cps2I_1/2-Fw | GATTTGTCGGGAGGGTTACTTG | 61 | 450 | [16] |
|  |  | cps2I_1/2-Rev | TAAATAATATGCCACTGTAGCGTCTC |  |  |  |
| *cps*14H-1I | *cps*14H and cps1I | cps14H_1I-Fw | AATCATGGAATAAAGCGGAGTACAG | 61 | 550 | [16] |
|  |  | cps14H_1I-Rev | ACAATTGATACGTCAAAATCCTCACC |  |  |  |
| *cps*2I-14K | cps2I and cps14K | cps2I_14K-Fw | CTTTGTGGTGGCCTGG | 61 | 209 | [16] |
|  |  | cps2I_14K-Rev | AATGGAAGCGATGGTCAG |  |  |  |
| *cps*3L | cps3L | cps3L-Fw | GGTTTTGATTGGTCTAGTTG | 58 | 214 | [17] |
|  |  | cps3L-Rev | CTCTAAAGCTCGATATCTAC |  |  |  |
| *cps*4K | cps4K | cps4K-Fw | GACTATCTGTATACCCAAAC | 54 | 903 | [17] |
|  |  | cps4K-Rev | TCCTTCCAAGTATTCTCTAG |  |  |  |
| *cps*5L | cps5L | cps5L-Fw | ATCTTAGGAATGATTCGGAC | 58 | 720 | [17] |
|  |  | cps5L-Rev | ACCAGATATCTGAGCAAATG |  |  |  |
| *cps*7L | cps7L | cps7L-Fw | AACTACCTACCTGAACTTTG | 58 | 566 | [17] |
|  |  | cps7L-Rev | AGTCTAAAAGTGATCGAGTC |  |  |  |
| *cps*8K | cps8K | cps8K-Fw | AAATAAGGTAGGAGCTACTC | 58 | 446 | [17] |
|  |  | cps8K-Rev | ATCCAACCTTAGCTTTCTGT |  |  |  |
| *cps*9J | cps9J | cps9J-Fw | GAAAGTAGGTATATCTCAGC | 58 | 368 | [17] |
|  |  | cps9J-Rev | GGGCTATTAAAACTCCTATC |  |  |  |
| **MLST** | | | |  | | |
| *dpr* | putative peroxide resistance protein | dpr-Fw | CGTCTTTCAGCCCGCGTCCA | 52 | 462 | [18] |
|  |  | dpr-Rev | GACCAAGTTCTGCCTGCAGC |  |  |  |
| *thrA* | aaspartokinase/homoserine dehydrogenase | thrA-Fw | GATTCAGAACGTCGCTTTGT | 52 | 575 | [18] |
|  |  | thrA-Rev | AAGTTTTCATAGAGGTCAGC |  |  |  |
|  |  | thrA-Seq* | AAGAATGGATCATCAACCGT | - | - |  |
| *cpn60* | 60KDa chaperonin | cpn60-Fw | TTGAAAAACGTRACKGCAGGTGC | 52 | 506 | [18] |
|  |  | cpn60-Rev | ACGTTGAAIGTACCACGAATC |  |  |  |
| *recA* | homologous recombination factor | recA-Fw | TATGATGAGTCAGGCCATG | 52 | 423 | [18] |
|  |  | recA-Rev | CGCTTAGCATTTTCAGAACC |  |  |  |
| *gki* | glucose kinase | gki-Fw | GGAGCCTATAACCTCAACTGG | 55 | 545 | [18] |
|  |  | gki-Rev | AAGAACGATGTAGGCAGGATT |  |  |  |
| *aroA* | 5 enolpyruvylshikimate 3-phosphate synthase | aroA-Fw | TTCCATGTGCTTGAGTCGCTA | 55 | 536 | [18] |
|  |  | aroA-Rev | ACGTGACCTACCTCCGTTGAC |  |  |  |
| *mutS* | DNA mismatch repair enzime | mutS-Fw | CGCAGAGCAGATGGAAGATCC | 55 | 454 | [19] |
|  |  | mutS-Rev | CCCATAGCTGTTTTGGTTTCATC |  |  |  |
| **Gene encoding virulence factors** | | | |  | | |
| *epf* | Extracellular factor | epf-Fw | CGCAGACAACCGAAAGATTGA | 58 | 744 | [20] |
|  |  | epf-Rev | AAGAATGTCTTTGGCGATGG |  |  |  |
| *sly* | Suilysin | sly-Fw | GCTTGACTTACGAGCCACAA | 58 | 248 | [20] |
|  |  | sly-Rev | CCGCGCAATACTGATAAGC |  |  |  |
| *mrp* | Muramidase-released preotein | mrp-Fw | ATTGCTCCACAAGAGGATGG | 58 | 188 | [20] |
|  |  | mrp-Rev | TGAGCTTTACCTGAAGCGGT |  |  |  |
| *hylA* | hyaluronidase | hylA-Fw | GATTTGGTCAGAAAAGGAATG | 55 | 796 | This study |
|  |  | hylA-Rev | TTGTTGACCAAATCAATATCCT |  |  |  |
| *dppIV* | Dipeptidyl-aminopeptidase IV | dppIV-Fw | ATGCGCTTTAATCAATTTTCT | 53 | 955 | This study |
|  |  | dppIV-Rev | GACCGTTCAGCCAGTCAA |  |  |  |
| *igA* | Specific zinc metalloproteinase | igA-Fw | GGAGTTGATTATTCGTGAC | 55 | 1076 | This study |
|  |  | igA-Rev | AGTTCTTCTGTTTCTGTG |  |  |  |
| *spb2* | putative pilus subunit protein | spb2-Fw | AAATTTACTTATGAAGAAAA | 48 | 393 | This study |
|  |  | spb2-Rev | GAGTTTTGGTATCAAAGTAT |  |  |  |
| *sspA* | subtilisin like serine proteinase | sspA -Fw | GGTTATAACTATGTTGATGTG | 55 | 715 | This study |
|  |  | sspA -Rev | CATTGGCTATTTTTTCTGAG |  |  |  |
| *apuA* | Surface anchored amylopullulanase | apuA -Fw | TTTGCCTTTACAGGTATGTA | 48 | 1150 | This study |
|  |  | apuA -Rev | CATTTTGACCTGGAATTGAAATC |  |  |  |
| SSU1773 | Surface anchored serine protease | SSU1773 -Fw | TCAATAAAGAAAGTCTTGGT | 55 | 619 | This study |
|  |  | SSU1773 -Rev | TTTAACCGTACCTTATATTTC |  |  |  |
| *htpsC* | Histidine triad protein C | htpsC -Fw | GGTTACTCATTGGCCATG | 55 | 608 | This study |
|  |  | htpsC -Rev | CTAACACTTCAACATTTGG |  |  |  |
| *ofs* | Serum opacity factor | ofs-Fw | GCTGAAAACACTAGTTCTT | 55 | 584 | This study |
|  |  | ofs-Rev | CTCCCCTTGTGACAGAAG |  |  |  |
| *srtF* | Sortase F | srtF-Fw | AGTGATTACTGGAATTCCTT | 55 | 477 | This study |
|  |  | srtF-Rev | ATGGCTCAACCACTCGAA |  |  |  |

* thrA-Seq primer was used for sequencing *thrA* gene fragment as described King, et al. [18].
